# Erythrocytosis due to *PHD2* Mutations: A Review of Clinical Presentation, Diagnosis, and Genetics

**DOI:** 10.1155/2016/6373706

**Published:** 2016-02-29

**Authors:** Rachel Wilson, Nausheen Syed, Prabodh Shah

**Affiliations:** ^1^Chicago Medical School, North Chicago, IL, USA; ^2^Little Company of Mary Hospital, Evergreen Park, IL, USA

## Abstract

The association of mutations in the PHD2 protein of the hypoxia-sensing pathway and erythrocytosis has only been established in the last decade. Here we report the case of a novel* PHD2* gene mutation in a patient with erythrocytosis and summarize all reported cases to date.* Case Report*. A 55-year-old man presented with dyspnea and a previous diagnosis of idiopathic erythrocytosis.* PHD* gene sequencing revealed a mutation on exon 2. The mutation was recognized as p.(Trp334^⁎^) (c. 1001G>A) resulting in a truncation of a highly conserved amino acid residue in catalytic domain. A diagnosis of erythrocytosis secondary to mutant* PHD2* gene was made.* Conclusions*. Our findings indicate that with* PHD2* mutations there is moderate erythrocytosis and erythropoietin (Epo) levels are generally low to normal. Two patients with* PHD2* substitution mutations were found to have paraganglioma and one of these patients had a concurrent pheochromocytoma. In addition, one mutation was associated with sagittal sinus thrombosis. Given the severity of some of the clinical features of these mutations, we conclude that clinical guidelines should include the* PHD2* mutation in the idiopathic erythrocytosis workup.

## 1. Introduction

The human body is intricately adapted to respond to low oxygen states. Hypoxia activates the hypoxia-inducible factor (HIF) pathway resulting in an increased production of erythropoietin (EPO) which, in turn, activates red blood cell production by binding receptors on erythroid progenitor cells. The HIF pathway includes the HIF-*α* transcription factor, the prolyl hydroxylase domain (PHD) protein family, and von Hippel-Lindau tumor suppressor protein (pVHL). There are three major isoforms of the PHD protein that are capable of binding HIF-*α*, but PHD2 has been shown to be the most critical protein taking part in the oxygen-sensing pathway [1]. Under conditions of normal oxygen tension, the PHD proteins hydroxylate HIF-*α* allowing for the binding of the pVHL protein. Upon binding HIF-*α*, pVHL forms an E3 ubiquitin ligase complex which allows for HIF-*α* ubiquitination and subsequent degradation by a proteasome. However, under hypoxic conditions, PHD2 reduces hydroxylation of HIF-*α*, allowing HIF-*α* to escape ubiquitin mediated degradation. Subsequently, HIF-*α* acts as a transcription factor and upregulates expression of* EPO* gene resulting in increased EPO production. EPO is released into the bloodstream, binds to the Epo receptor (EpoR), and promotes growth and differentiation of erythroid precursors in the bone marrow. This causes an increase in red blood cell mass to compensate for the hypoxic condition.

The association of hereditary erythrocytosis with mutations of the* PHD2* gene (also known as the* EGLN1* gene) has been previously reported [2]. The erythrocytosis occurs by the decreased binding ability of the PHD2 protein and subsequent increased activity of HIF-*α* [3]. Upregulation of HIF-*α* increases red cell mass and may contribute to vascular proliferation and cell growth [4]. This can lead to complications such as pulmonary hypertension and thrombophlebitis [5]. There are also likely decreased interactions of the PHD2 protein with other binding proteins that may contribute to erythropoiesis [6].

We report a patient with a novel* PHD2* gene mutation, p.(Trp334^*∗*^), and review the literature with described* PHD2* gene mutations.

Our patient presented to our clinic in 2014 at the age of 55 with recent onset of mild dyspnea and a diagnosis of erythrocytosis since 2009. His medical history included hypertension, hyperlipidemia, sleep apnea, atrial fibrillation, and type 2 diabetes mellitus. His medications included furosemide, enalapril, simvastatin, warfarin, aspirin, digoxin, and metformin. He was a nonsmoker. He was treated with phlebotomies in the past and his hemoglobin values had fluctuated from 180 g/L to 215 g/L. His sister had hemoglobin of 162 g/L. Otherwise family history was unremarkable.

Physical exam included temperature of 36.9°C, blood pressure of 127/79 mmHg, pulse rate of 60 beats per minute, and respiratory rate of 12 breaths per minute. Findings included a mildly ruddy complexion, lungs clear to auscultation bilaterally, and irregular heart tones. There was neither hepatosplenomegaly nor masses. Extremities revealed no edema and lymph node exam was unremarkable. Neurological exam was normal.

Investigations showed red cells count was 7.41 × 10^12^/L, hemoglobin was 194 g/L, white blood cell count was 6.5 × 10^9^/L, and platelet count was 255 × 10^9^/L. The oxygen saturation was 98%, nocturnal oxygen saturation remained above 92%, carboxyhemoglobin was 2.3%, and no abnormal hemoglobin was detected on electrophoresis or isoelectrofocusing. The P_50_ was 28 mmHg (24–30 mmHg) and erythropoietin was 8.3 IU/L (4.0–16.0 IU/L). CT scan did not show splenomegaly or intra-abdominal masses suspicious for tumors.

Bone marrow aspirate showed normal maturation and iron stores were present. Cytogenetic analysis did not reveal any abnormal karyotype. *JAK*2^V617F^ mutation was not identified on exon 14; neither were* JAK2* mutations found for exons 8 or 12.* HIF-2α (EPAS1)* and* EPOR* gene sequencing did not reveal any mutations. However,* PHD* gene sequencing revealed a mutation on exon 2. The mutation was recognized as p.(Trp334^*∗*^) (c. 1001G>A). A diagnosis of erythrocytosis secondary to mutant* PHD2* gene was made.

Currently, the patient remains asymptomatic.

## 2. Discussion

Patients included in this review had erythrocytosis and mutations of the* PHD2* gene resulting in loss of function of PHD2 proteins. Familial cases suggest autosomal dominant inheritance whereas the isolated cases are likely sporadic germline mutations. There are twenty-five different mutations reported of the* PHD2* gene: fifteen substitutions, five nonsense mutations, and five frameshift mutations. Most mutations involve evolutionarily conserved residues that influence protein catalytic activity (see Table 1).

In the HIF pathway, erythrocytosis associated with HIF and VHL proteins is associated with high EPO levels [7]. However, our findings indicate that, with* PHD2* gene mutations, EPO levels are generally low to normal. In our review, twenty-one patients had normal EPO levels, four patients had unexplained elevated levels, two had a low level, and the rest were unknown. The low-normal EPO level in the face of erythrocytosis in* PHD2* mutants likely involves heightened expression and sensitivity of EPO receptor and HIF regulation [8]. The heightened EpoR sensitivity and expression are presumably a result of PHD2's regulation of HIF and PHD2's interaction with other binding proteins besides HIF [6].

The association of tumorigenesis and germline mutations of proteins in hypoxia-sensing pathway has already been well established [9, 10]. The most well-known syndrome to associate erythrocytosis and tumorigenesis is von Hippel Lindau disease, but mutations in* PHD1* and* HIF2a (EPAS1)* are also implicated in tumorigenesis [8, 11]. In our review, we found two patients with* PHD2* substitution mutations who had paragangliomas and one of these patients had a concurrent pheochromocytoma [8, 12]. Furthermore,* PHD2* germline mutations can have oncogenic potential in vitro [13] and analysis of paraganglioma cells suggests* PHD2* may have tumor suppressor activity since the tumor cells showed loss of heterozygosity of wild-type* PHD2* allele [6]. Suppression of tumor growth may be related to the* PHD2* gene's regulation of various cytokines [14]. These observations suggest that therapeutic guidelines regarding the diagnostic workup of idiopathic erythrocytosis need to consider the possibility of these autosomal dominant mutations and may benefit the family members by providing early detection of tumors. In addition, the association of a* PHD2* substitution mutation and sagittal sinus thrombosis warrants search for thrombotic risks of such mutations [3].

## Figures and Tables

**Table 1:** Characteristics of patients with *PHD2* mutations and polycythemia.

Number	Age/sex^∖∖^	Hb (g/L)	EPO (IU/L)	Relevant clinical data	Mode of inheritance^¶^	Associated mutations	Relation to protein structure	Protein changes and mutation (catalytic domain from amino acid residues 181–426 [15])	Reference
1	43 (44) M	202	24.0 (5–25)	N/A	N/A	Homozygous *JAK2* V617F	Substitution between N-terminal MYND zinc finger-like domain and conserved C-terminal catalytic domain	p.(Gln157His); c.471G>C	Ladroue et al. [6]

2	65 (65) M	171	29.0 (5–30)	Thrombocytosis, leukocytosis and splenomegaly, Phlebotomies, and hydroxycarbamide	Autosomal dominant	Homozygous *JAK2* V617F	Substitution between N-terminal MYND zinc finger-like domain and conserved C-terminal catalytic domain	p.(Gln157His); c.471G>C	Albiero et al. [16]

3	40 (40) M	170	8.0 (5–30)	N/A	Autosomal dominant	None	Substitution between N-terminal MYND zinc finger-like domain and conserved C-terminal catalytic domain	p.(Gln157His); c.471G>C	Albiero et al. [16]

4	22 (34) M	179	90.0 (5–25)	N/A	N/A	None	Substitution of highly conserved amino acid one residue from site that chelates Zn and Cd ions Mutation causes delayed hydroxylation of HIF-*α*	p.(Pro200Gln); c.599C>A	Ladroue et al. [6]

5	54 (54) M	192	20.0 (5–25)	Inflammatory arthromyalgia, visual symptoms, and phlebotomies	Autosomal dominant	None	Truncation in catalytic domain of 154 C-terminal amino acids	p.(Met202Ilefs^*∗*^72); c.606delG	Al-Sheikh et al. [17]

6	>54 (>54) M	171	11.5 (5–25)	Phlebotomies	Autosomal dominant	None	Truncation in catalytic domain of 154 C-terminal amino acids	p.(Met202Ilefs^*∗*^72); c.606delG	Al-Sheikh et al. [17]

7	61 (80) M	230	2.0 (5–25)	Hemorrhage, phlebotomy, and aspirin	N/A	*JAK2*-exon 12	Substitution of highly conserved part of catalytic site	p.(Asn203Lys); c.609C>G	Albiero et al. [18]

8	49 (46) M	200	11.0 (5–25)	Cardiac disease	N/A	None	Substitution mutation of catalytic site	p.(Lys204Glu); c.610G>A	Bento et al. [19]

9	52 (51) M	183	8.13 (5–25)	Klinefelter's syndrome	N/A	None	Truncation mutation of catalytic site	p.(Gln221^*∗*^); c.661C>T	Lambert, unpublished data (2013)^§^

10	34 (24) M	172	N/A	Headaches	N/A	None	Truncation mutation of catalytic site	p.(Arg227Alafs^*∗*^20); c.678dupG	Bento and Almeida, unpublished data (2014)^§^

11	16 (60) F	160	40.5 (<31.5)	Red eyes, flushed cheeks and feet headache, episodic chest pain palpitations, and primary hyperparathyroidism cystic kidney disease paraganglioma pheochromocytoma repeated phlebotomies	N/A	None	Substitution of highly conserved residue site likely to affect protein folding and stability	p.(Ala228Ser); c.682G>T	Yang et al. [8]

12	52 (58) M	178	N/A		N/A	None	Truncation mutation of catalytic site	p.(Gln239^^*∗*^^); c.715C>T	Bento and Almeida, unpublished data (2014)^§^

13	25 (48) M	192	2x normal (5–25)	N/A	N/A	None	Substitution of highly conserved residue of catalytic site	p.(Asp254His); c.760G>C	Ladroue et al. [6]

14	73 (73) M	188	1.3 (10.2–28.5)	Smoker	N/A	None	Loss of catalytic activity of PHD2 protein	p.(Leu279Thrfs43^*∗*^); c.835del14	Jang et al. [20]

15	22 (22) M	178	N/A	Tinnitus	N/A	None	Truncation of 143 C-terminal amino acids	p.(Arg281Thrfs^*∗*^3); c.840_841insA	Al-Sheikh et al. [17]

16	68 (65) M	183	60 (5–25)		N/A	None	Substitution mutation of catalytic site	p.(Gly285Arg); c.853G>C	Bento et al. [19]

17	29 (38) M	176	5.0 (5–25)	N/A	Autosomal dominant	None	Substitution of nonconserved residue of catalytic domain	p.(Lys291Ile); c.872A>T	Albiero et al. [18]

18	48 (48) F	180	6.2 (5–25)	Leukoclastic vasculitis	N/A	None	Substitution mutation of catalytic site	p.(Pro304Leu); c.911C>T	Percy and McMullin, unpublished data (2004)^§^

19	45 (45) M	180	N/A	Smoker with intermittent claudication and death from esophageal carcinoma	Autosomal dominant	None	Substitution of highly conserved amino acid, close proximity to site responsible for coordinating Fe^2+^ at active site	p.(Pro317Arg); c.950C>G	Percy et al. [2]

20	26 (26) F	180	6.3 (5–25)	Superficial thrombophlebitis, history of menorrhagia, and phlebotomies	Autosomal dominant	None	Substitution of highly conserved amino acid, close proximity to site responsible for coordinating Fe^2+^ at active site	p.(Pro317Arg); c.950C>G	Percy et al. [2]

21	30 (30) M	175	6.4 (5–25)	Paresthesia, absent left kidney, and enlarged right kidney	Autosomal dominant	None	Substitution of highly conserved amino acid, close proximity to site responsible for coordinating Fe^2+^ at active site	p.(Pro317Arg); c.950C>G	Percy et al. [2]

22	31 (31) F	174	6.0 (3–34)	N/A	Autosomal dominant	None	Substitution of highly conserved amino acid of catalytic domain	p.(Trp334Arg); c.1000T>C	Bento et al. [21]

23	49 (55) M	215	8.3 (4–16)	Phlebotomies	Sister has polycythemia	None	Truncation in highly conserved amino acid residue in catalytic domain	p.(Trp334^*∗*^); c.1001G>A	(this paper)

24	35 (35) F	178	10.7 (5–25)	Phlebotomies	N/A	None	Truncation of 50 C-terminal amino acids	p.(Gln337^*∗*^); c.1129C>T	Al-Sheikh et al. [17]

25	21 (24) M	171	9.9 (5–25)	TIA	N/A	None	Truncation mutation of catalytic site	p.(Val338Glyfs^*∗*^18); c.1010dup	Bento et al. [19]

26	47 (47) M	168	9.5 (5–25)		N/A	None	Substitution mutation of catalytic site	p.(Arg371Cys); c.1111C>T	Percy and McMullin, unpublished data (2013)^§^

27	17 (25) M	191	Normal (5–25)	N/A	N/A	None	Substitution of highly conserved amino acid 3 residues away from Fe^2+^ chelating residue	p.(Arg371His); c.1112G>A	Ladroue et al. [6]

28	29 (38) M	188	12.0 (5–25)	Sagittal sinus thrombosis and phlebotomies	N/A	None	Substitution of highly conserved amino acid 3 residues away from Fe^2+^ chelating residue	p.(Arg371His); c.1112G>A	Percy et al. [3]

29	30 (43) M	202	18.0 (5–25)	Recurrent para-aortic paraganglioma hypertension phlebotomies	N/A	Homozygous *C282Y* mutation	Substitution of highly conserved amino acid critical to coordinating Fe^2+^ binding	p.(His374Arg); c.1121A>G	Ladroue et al. [12]

30	64 (67) F	161	N/A	Suspected liver and renal angiomas	Autosomal dominant	None	Truncation in catalytic domain	p.(Arg398^*∗*^); c.1192C>T	Ladroue et al. [6]

31	26 (41) M	193	6.5 (5–25)	N/A	Autosomal dominant	None	Truncation in catalytic domain	p.(Arg398^*∗*^); c.1192C>T	Ladroue et al. [6]

32	60 (80) M	164	23.0 (5–25)	Treated with aspirin and phlebotomies	N/A	None	Substitution of highly conserved residue mutation of catalytic domain	p.(Lys423Glu); c.1267A>G	Albiero et al. [18]

^∖∖^Age at diagnosis (age at workup for mutation).

^§^Original sources listed, data later compiled in review by Gardie et al. [22]

^¶^Mode of inheritance determined by family history.
